# Nutrition-Related Policy and Environmental Strategies to Prevent Obesity in Rural Communities: A Systematic Review of the Literature, 2002–2013

**DOI:** 10.5888/pcd12.140540

**Published:** 2015-04-30

**Authors:** Larissa Calancie, Jennifer Leeman, Stephanie B. Jilcott Pitts, Laura Kettel Khan, Sheila Fleischhacker, Kelly R. Evenson, Michelle Schreiner, Carmen Byker, Clint Owens, Jared McGuirt, Ellen Barnidge, Wesley Dean, Donna Johnson, Jane Kolodinsky, Emily Piltch, Courtney Pinard, Emilee Quinn, Lauren Whetstone, Alice Ammerman

**Affiliations:** Author Affiliations: Jennifer Leeman, Kelly R. Evenson, Michelle Schreiner, Clint Owens, Jared McGuirt, Alice Ammerman, University of North Carolina at Chapel Hill, Chapel Hill, North Carolina; Stephanie B. Jilcott Pitts, Lauren Whetstone, East Carolina University, Elizabeth City, North Carolina; Laura Kettel Khan, Centers for Disease Control and Prevention, Division of Nutrition, Physical Activity, and Obesity, Atlanta, Georgia; Sheila Fleischhacker, National Institutes of Health, Division on Nutrition Research Coordination, Bethesda, Maryland; Carmen Byker, Montana State University, Billings, Montana; Ellen Barnidge, Saint Louis University, St. Louis, Missouri; Wesley Dean, US Department of Agriculture Food and Nutrition Service, Washington, DC; Donna Johnson, Emilee Quinn, University of Washington, Seattle, Washington; Jane Kolodinsky, University of Vermont, Burlington, Vermont; Emily Piltch, Tufts University, Boston, Massachusetts; Courtney Pinard, University of Nebraska, Lincoln, Nebraska.

## Abstract

**Introduction:**

Residents of rural communities in the United States are at higher risk for obesity than their urban and suburban counterparts. Policy and environmental-change strategies supporting healthier dietary intake can prevent obesity and promote health equity. Evidence in support of these strategies is based largely on urban and suburban studies; little is known about use of these strategies in rural communities. The purpose of this review was to synthesize available evidence on the adaptation, implementation, and effectiveness of policy and environmental obesity-prevention strategies in rural settings.

**Methods:**

The review was guided by a list of Centers for Disease Control and Prevention *Recommended* *Community Strategies and Measurements to Prevent Obesity in the United States,* commonly known as the “COCOMO” strategies. We searched PubMed, Cumulative Index of Nursing and Allied Health Literature, Public Affairs Information Service, and Cochrane databases for articles published from 2002 through 2013 that reported findings from research on nutrition-related policy and environmental strategies in rural communities in the United States and Canada. Two researchers independently abstracted data from each article, and resolved discrepancies by consensus.

**Results:**

Of the 663 articles retrieved, 33 met inclusion criteria. The interventions most commonly focused on increasing access to more nutritious foods and beverages or decreasing access to less nutritious options. Rural adaptations included accommodating distance to food sources, tailoring to local food cultures, and building community partnerships.

**Conclusions:**

Findings from this literature review provide guidance on adapting and implementing policy and environmental strategies in rural communities.

## Introduction

Residents of rural communities in the United States experience disproportionately high rates of obesity and other nutrition-related chronic diseases than do urban and suburban residents (1–3). Addressing rural health disparities is a key objective of *Healthy People 2020* (4). Research suggests that less healthy eating patterns contribute to these disparities (5). Poverty in rural areas and a paucity of healthy retail food outlets limit access to healthy foods and contribute to less healthy diets (5–7). Policy and environmental strategies (eg, zoning policies that facilitate the location of farmers markets in underserved areas) can help increase access to healthy foods and beverages and thereby increase opportunities for making healthy food choices (8–10). Additional advantages of strategies that target change at the levels of policy and environment include lower per-person costs and greater potential for long-term sustainability than strategies that target change at the individual level (8,11).

The evidence in support of nutrition-related policy and environmental strategies is based largely on urban and suburban studies; thus, little is known about their use in rural communities. Rural communities may have distinct cultures and infrastructures that limit the transferability of strategies from nonrural contexts (12–15). Rural areas may also lack financial and human resources necessary to adopt and implement policy and environmental changes that work in an urban context. Still, rural areas may have assets, such as greater collaboration across public and private sectors, which may lead to strong obesity prevention partnerships (15).

The purpose of this study was to synthesize the evidence on the adoption, implementation, and effectiveness of nutrition-related policy and environmental obesity-prevention strategies in rural settings. The review was guided by the Centers for Disease Control and Prevention’s (CDC) *Recommended Community Strategies and Measurements to Prevent Obesity in the United States,* commonly known as the “COCOMO” strategies (16), which are widely used in public health (17). This study focused on COCOMO’s 10 nutrition-related strategies (Table 1). Our a priori hypothesis was that some but not all of the COCOMO strategies had been implemented in rural areas and that implementation required adaptations to the rural context.

**Table 1:** Ten Nutrition-Related Strategies from Centers for Disease Control and Prevention’s *Recommended* *Community Strategies and Measurements to Prevent Obesity in the United States* (16)

Strategy Number	Strategy Description
1	Increase availability of healthier food and beverage choices in public service venues.
2	Improve availability of affordable healthier food and beverage choices in public service venues.
3	Improve geographic availability of supermarkets in underserved areas.
4	Provide incentives to food retailers to locate in and/or offer healthier food and beverage choices in underserved areas.
5	Improve availability of mechanisms for purchasing foods from farms.
6	Provide incentives for the production, distribution, and procurement of foods from local farms.
7	Restrict availability of less healthy foods and beverages in public service venues.
8	Institute smaller portion size options in public service venues.
9	Limit advertisements of less healthy foods and beverages.
10	Discourage consumption of sugar-sweetened beverages.

## Methods

We conducted a systematic review of the literature to identify, extract, and integrate findings from empirical research on the use of nutrition-related policy and environmental strategies for obesity prevention in rural communities. The review was conducted by members of the Rural Food Access Work Group of the CDC-funded Nutrition and Obesity Policy Research and Evaluation Network (NOPREN), a nationwide network of more than 15 funded and affiliated partners that identifies and prioritizes a policy research agenda to improve access to healthy, affordable foods in rural communities (6). This project included the Policy Identification, Policy Evaluation, and Translation, Communication, and Dissemination of Research concepts from the NOPREN policy continuum (18).

### Data sources

PubMed, Cumulative Index of Nursing and Allied Health Literature, Public Affairs Information Service, and Cochrane databases were searched for articles published between January 1, 2002, and June 30, 2013, in English, that reported findings from formative, process, or outcome research on nutrition-related policy and environmental strategies in rural settings. To be comprehensive and capture strategies in addition to those of COCOMO, we searched broadly for nutrition-related policy and environmental strategies applied to obesity prevention. Each search was conducted by using the following terms: rural AND (nutrition or food) AND (community or environment or policy) AND (obesity or overweight or “chronic disease”). In addition to using the search term “rural,” the search was repeated in each database by using predominantly rural states as search terms. The predominantly rural states were identified using the Rural-to-Urban Continuum Codes, the Office of Management and Budget maps, or the Rural Assistance Center’s Frontier map where substantial portions of the state are frontier. The search included relevant references cited in each of the identified studies and in prior reviews of the literature on nutrition-related policy and environmental strategies. NOPREN colleagues also recommended relevant articles.

### Study selection

At least 2 members of the research team screened titles and abstracts and then reviewed the complete text of relevant articles to select articles for inclusion. To be included, the article had to report findings from empirical formative, process, or outcome research related to policy or environmental obesity-prevention strategies in rural communities in the United States or Canada. The term “rural” was broadly defined so as to allow for inclusion of any study in which authors described the setting as “rural,” “non-metro,” “small town,” or “remote” or a study conducted in counties that the Health Resources and Services Administration characterized as rural in 2005 (19). Policy and environmental strategies included, but were not limited to, the 10 nutrition-related COCOMO strategies (Table 1). Although the original COCOMO strategies applied to public service venues, for this study’s purpose COCOMO strategies were expanded to apply to any setting (eg, worksites). Articles that included both rural and urban communities were included only if they reported rural-specific findings.

Data were abstracted from each article by using a standardized form. The form included information about study population (eg, race/ethnicity, socioeconomic status), setting, geographic location, approaches used to adapt the intervention or its implementation to a rural setting, design, methods, and findings. All 17 data abstractors were trained using a strategy similar to that employed by the US Department of Agriculture (USDA) Center for Nutrition Policy and Promotion Nutrition Evidence Library (20). Similar to the USDA’s process, 2 members of the team independently abstracted data, compared abstractions, and then resolved discrepancies by consensus for each article.

Data from the consensus abstraction forms were integrated using data matrices. Four members of the research team reviewed the matrices to identify themes, and tables and narratives were created summarizing data related to those themes.

## Results

The search identified 663 articles, and 33 articles (reporting the findings from 29 studies) met inclusion criteria after exclusions (Figure) (Table 2). Findings are reported as follows: 1) study locations, settings, and study approach; 2) types of policy and environmental obesity prevention strategies used; 3) approaches to adapting and implementing nutrition-related policy and environmental strategies for obesity prevention in rural areas; and 4) intervention effects on policy, environment, behavioral, and health outcomes (as a part of Policy Evaluation).

**Table 2:** Citation, Geographic Location, Setting(s), and Evaluation Type for Studies of Nutrition-Related Policy and Environmental Strategies for Obesity Prevention Conducted in Rural Areas of the United States and Canada, 2002–2013

Citation	Geographic Location	Setting(s)	Evaluation Type
Bachar et al, 2006 (21)	Reservations, Western, North Carolina	Worksites, faith-based institutions, community	Process, outcome
Belansky et al, 2010 (22)	Colorado	Schools	Process, outcome
Brown et al, 2010 (23)	Reservations, Montana	Schools, small retail food outlets	Formative
Conrey et al, 2003 (24)	New York	Farmers markets	Outcome
Curran et al, 2005 (25)	Reservations, Arizona	Small retail food outlets, community	Process
Drummond et al, 2009 (26)	Yuma County, Arizona	Child care	Outcome
Escoffery et al, 2011 (27)	Southwest Georgia	Worksites	Formative
Flamm, 2011 (28)	Ohio	Farmers markets	Formative
Fleischhacker et al, 2012 (29)	American Indian tribes in North Carolina	Community	Formative
Gittelsohn et al, 2010 (30)	First Nations, Nunavut, Canada	Small retail food outlets	Formative
Gombosi, 2007 (31)	Tioga County, Pennsylvania	Schools, community, worksites	Outcome
Harris et al, 2010 (32)	West Virginia	Schools	Process
Ho et al, 2006 and 2008 (33,34)	First Nations, Ontario, Canada,	Schools, small retail food outlets	Formative, outcome
Johnston et al, 2009 (35)	Broome County and Tioga County, New York	Schools	Outcome
Knol et al, 2010 (36)	Southeastern United States	Health facilities	Outcome
Kunkel et al, 2003 (37)	South Carolina	Farmers markets	Outcome
Laing et al, 2012 (38)	Mason County, Washington	Worksites	Process, outcome
Mead et al, 2010 and 2013 (39,40)	First Nation, Canadian Arctic	Small retail food outlets, community	Formative, outcome
Nanney et al, 2008 (41)	Utah	Schools	Process
Novotny et al, 2011 (42)	Hawaii	Small retail food outlets, community	Process
O’Brien et al, 2010 (43)	Maine	Schools	Outcome
Phillips et al, 2013 (44); Raczynski et al, 2009 (45)	Arkansas	Schools	Process, outcome
Rosecrans et al, 2008 (46); Saksvig et al, 2005 (47)	First Nation, Ontario, Canada	Small retail food outlets, community, schools	Process, outcome
Ruelle et al, 2011 (48)	Reservations, North Dakota and South Dakota	Farmers markets	Process
Schetzina et al, 2009 (49)	Northeast Tennessee	Schools	Formative
Schwarte et al, 2010 (50)	California Central Valley	Community, worksites, schools, public health	Process
Setala et al, 2011 (51)	Reservations, Arizona, Utah, New Mexico	Small retail food outlets, farmers markets	Formative
Sussman and Davis, 2010 (52)	New Mexico	Schools, small retail food outlets, community	Formative
Vastine et al, 2005 (53)	Reservations, Arizona	Small retail food outlets	Formative

**Figure:**
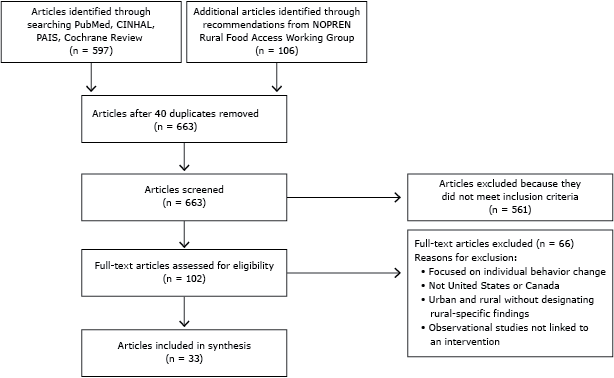
Preferred Reporting Items for Systematic Reviews and Meta-Analysis (PRISMA) flow diagram for study inclusion is a systematic review of nutrition-related policy and environmental strategies for obesity prevention applied in rural communities. Abbreviations: CINAHL, Cumulative Index of Nursing and Allied Health Literature; PAIS, Public Affairs Information Service; NOPREN, Nutrition and Obesity Policy Research and Evaluation Network.

### 1. Study locations, settings, and approach

Of the 29 studies included, 4 took place in Canada (14%) and 25 in the United States (86%) (Table 2). Approximately one-third of the studies (n = 10, 34%) were conducted with American Indian tribes or with First Nations of Canada. The most common settings were schools (n = 13, 45%), small retail food outlets (n = 10, 34%), worksites (n = 5, 17%), and farmers markets (n = 5, 17%). Small retail food outlets were the setting for 70% of studies with American Indian tribes or First Nations (n = 7). About one-third (n = 11, 37%) of the studies took place in multiple settings. Half of the studies (n = 15, 52%) reported findings from formative or process evaluations and did not include outcome data. Fourteen studies (48%) reported findings from an outcome evaluation.

### 2. Types of nutrition-related policy and environmental strategies used

The COCOMO strategy used most often was strategy 1, “increase availability of healthier food and beverage choices” (Table 3). That is, strategy 1 was used in 22 studies (76%), followed by strategy 7, “restrict availability of less healthy foods and beverages” (n = 11, 38%). The strategies used least frequently were strategy 8, “institute smaller portion size options in public service venues” (n = 1, 3%), and strategy 9 “limit advertisements of less healthy foods and beverages” (n = 1, 3%). None of the studies sought to improve the geographic availability of supermarkets (strategy 3).

**Table 3:** CDC Nutrition-Related Strategies^a^ Applied in Policy, Environmental, and Community-Level Intervention Studies Conducted in Rural Settings and Approaches for Adapting and Implementing Strategies in Rural Settings, 2002–2013

	COCOMO Strategy Applied											Approaches to Adapting and Implementing Obesity Prevention Strategies in Rural Areas		
Citation	1	2	3	4	5	6	7	8	9	10	NS	Accommodate Distance^b^	Tailor to Culture^c^	Build Partnerships^d^
Bachar et al, 2006 (21)	x									x				
Belansky et al, 2010 (22)	x						x			x				
Brown et al, 2010 (23)	x	x		x								x		
Conrey et al, 2003 (24)					x	x								x
Curran et al, 2005 (25)	x											x		
Drummond et al, 2009 (26)	x						x							
Escoffery et al, 2011 (27)	x	x					x					x		
Flamm et al, 2011 (28)					x	x								x
Fleischhacker et al, 2012 (29)				x									x	
Gittelsohn et al, 2010 (30)	x	x										x	x	
Gombosi et al, 2007 (31)	x													
Harris et al, 2010 (32)	x						x			x				
Ho et al, 2006 and 2008 (33,34)	x	x					x			x				
Johnston et al, 2009 (35)	x					x	x							
Knol et al, 2010 (36)	x						x	x		x				
Kunkel et al, 2003 (37)					x							x		
Laing et al, 2012 (38)											x			
Mead et al, 2010 and 2013 (39,40)	x	x										x	x	
Nanney et al, 2008 (41)	x													
Novotny et al, 2011 (42)	x				x	x						x		
O’Brien et al, 2010 (43)	x						x			x				
Phillips et al, 2013 (44); Raczynski et al, 2009 (45)	x						x		x	x				x
Rosecrans et al, 2008 (46); Saksvig et al, 2005 (47)	x													
Ruelle et al, 2011 (48)					x	x							x	
Schetzina et al, 2009 (49)	x						x							
Schwarte et al, 2010 (50)	x				x	x	x							
Setala et al, 2011 (51)					x	x								
Sussman and Davis et al, 2010 (52)	x	x		x										
Vastine et al, 2005 (53)	x	x										x	x	

Abbreviation: CDC, Centers for Disease Control and Prevention; COCOMO, Recommended Community Strategies and Measurements to Prevent Obesity in the United States; NS, not specified.

a From CDC’s *Recommended* *Community Strategies and Measurements to Prevent Obesity in the United* *States* (16).

b Accommodate long distances to food sources.

c Tailor strategies to distinct cultures and food preferences.

d Build strong local partnerships when implementing strategies.

### 3. Approaches to adapting and implementing nutrition-related policy and environmental strategies in rural areas

The literature reviewed yielded 3 themes related to strategy adaptation and implementation in rural communities (Table 3).

**Accommodate long distances to food sources when implementing strategies.** In 11 studies, investigators discussed how the design and implementation of interventions in rural communities accommodated long distances between food suppliers and retailers and between retailers and consumers. For example, several studies noted that long distances can increase food costs and limit the availability of foods that have limited shelf lives or are sensitive to long transit times (30,39,42). As a result, stores involved in store-based interventions may have trouble stocking the foods promoted through the intervention (40). These challenges are compounded when communities are remote and may rely on specialized transportation, such as annual barge deliveries or food mail programs as seen in a First Nations community in the Canadian Arctic (39). Efforts to address these challenges include strengthening networks between food producers, distributors, and retail food outlets (42). Adaptations also may be required to reduce the distance customers need to travel from their residences to grocery stores and farmers markets (15,23,53) or from worksites to restaurants serving healthy foods or other retail food options (27). For example, farmers markets may increase access by changing the locations where they sell produce (24,37).

**Tailor strategies to distinct cultures and food preferences.** Investigators of 5 studies identified the need to adapt rural interventions to include specific types of foods. For example, 3 studies reported integrating traditional foods into intervention materials (30,39,48). Another study reported on the importance of understanding cultural values and practices, such as Southern approaches to food preparation (54). The importance of culture was particularly salient in the research conducted with American Indian tribes. For example, 1 study highlighted the importance of engaging tribal leaders, recognizing the history of relationships among tribes, and identifying tribe-specific governance structures, priorities, resources, and champions (29).

**Build strong local partnerships when implementing strategies.** In 3 studies, investigators noted the importance of partnerships with groups that assist with the redemption of federal food and nutrition assistance program benefits, such as the Agricultural Extension Service (15,24) and Electronic Benefit Transfer administration organizations (28), and parks and recreation departments, hospitals, and health departments (44). Although strong local partnerships are often beneficial in suburban and urban settings, partnerships may be particularly important to leveraging limited resources in rural settings. Also, partnerships may naturally develop in rural communities where social and professional networks are likely to overlap at times because of small populations (55).

### 4. Intervention effects on policy, environment, behavioral, and health outcomes

Sixteen studies included data on the effectiveness of nutrition-related policy and environmental strategies (Table 4). Most studies (n = 11, 38%) used a quasi-experimental pretest/posttest design with no comparison group. Studies were conducted in 9 settings (communities, health facilities, schools, worksites, faith institutions, farmers markets, small stores, restaurants, and public health departments); some studies occurred in multiple settings.

**Table 4:** Description of Articles Reporting Policy and Environmental, Psychosocial, Behavioral, or Biological Outcomes After Implementing Nutrition-Related Policy and Environmental Strategies For Obesity Prevention in Rural Communities, 2002–2012

Citation	Design	Sample Size, Settings if Reported	Policy and Environment Change	Psychosocial Change	Behavioral Change	Biological Change
Bachar et al, 2006 (21)	Pretest–posttest, no comparison	1 school, up to 600 students	Increased availability of fruits and vegetables in school cafeterias	Improved knowledge about how to make healthier food choices among school children	—	—
Belansky et al, 2010 (22)	Pretest–posttest, no comparison	45 schools	Increased number of schools with nutrition-related policies	—	—	—
Conrey et al, 2003 (24)	Time series, no comparison	All New York State FMNP participants	—	—	Increased redemption of FMNP coupons used to purchase produce at farmers markets	—
Drummond et al, 2009 (26)	Pretest–posttest, no comparison	17 child care centers	Increased number of child care centers with nutrition-related policies and environmental changes	—	—	—
Gombosi et al, 2007 (31)	Pretest–posttest, nonrandomized comparison	9 restaurants, approximately 4,200 students in 3 school districts and 2 private schools	9 restaurants initiated menu labeling	—	—	BMI increased less among children in intervention versus comparison community
Ho et al, 2008 (34)	Pretest–posttest, no comparison	4 communities, 95 community members		Higher food acquisition and intention scores but not for food preparation, self-efficacy, or outcome expectancies	—	Weight status not changed
Johnston et al, 2009 (35)	Pretest–posttest, no comparison	15 school districts, up to 40,000 students	Schools more consistently complied with existing policy limiting calories from fat and saturated fat in school meals	More parents perceived school lunches as nutritious at posttest compared with pretest	Increased purchases of fresh fruits and vegetables; 3% increase in participation of school meal programs	—
Knol et al, 2010 (36)	Pretest–posttest, no comparison	5 transitional group homes for clients with mental illness; 65 clients	Group homes implemented policies about food options available in vending machine and cafeterias	—	—	Weight loss among most overweight and obese residents
Kunkel, 2003 (37)	Postsurvey	Unspecified number of farmers markets, 658 seniors participating in SFMNP in South Carolina	Farmers markets increased use of SFMNP	Increased intentions to eat fruits and vegetables year round, food preparation knowledge, and purchases of produce they had never tried before	—	—
Laing et al, 2012 (38)	Pretest–posttest, no comparison	23 worksites	Increase in number of worksites with a health-related policy	—	—	—
Mead et al, 2013 (40)	Pretest–posttest, non-randomized comparison	4 communities, 133 to 246 community members	—	Increased knowledge, self-efficacy, and intentions related to healthy foods among intervention participants compared with control group; decrease in healthy and unhealthy food acquisition scores	—	No change in BMI
O'Brien et al, 2010 (43)	Cross sectional	123 intervention schools, 205 control schools; 80,428 students	Increased number of schools with nutrition-related policies; increased odds of having healthy foods available at school events	—	Reduced odds of students drinking more than 2 sodas per week	—
Phillips et al, 2013 (44)	Pretest–posttest, no comparison	All public schools in the state; number ranged from 113 to 496 per school	Increased availability of healthy versus unhealthy foods and beverages available in schools	—	Reduced purchasing of beverages from vending machines among adolescents with access to vending machines; no change in reported soda consumption	—
Raczynski et al, 2009 (45)	Pretest–posttest, no comparison	Statewide policy	Increased number of schools with nutrition-related policies and increased availability of healthy versus unhealthy foods and beverages	—	—	Percentage of overweight and obese children remained stable after the policy went into place
Saksvig et al, 2005 (47)	Pretest–posttest, no comparison	1 school, 122 students	School initiated a policy banning high-fat and high-sugar snack foods; initiated a school breakfast program	Improved dietary knowledge, intention, self-efficacy	Decreased percentage of energy from fat among boys, not girls; Increased fiber intake, especially among those participating in school breakfast program	BMI and percent body fat increased

Abbreviation: —, not measured; BMI, body mass index; FMNP, Farmers Market Nutrition Program; SFMNP, Senior Farmers Market Nutrition Program.

Twelve of the studies (41%) reporting outcomes documented healthier food environments and policies following the intervention in schools (n = 7, 24%), health facilities (n = 1, 3%), child care centers (n = 1, 3%), restaurants (n = 1, 3%), farmers markets (n = 1, 3%), and worksites (n = 1, 3%).

Ten studies included interventions’ effects on health behaviors or theoretical constructs that are predictive of those behaviors (Table 4). Though results were mixed, interventions tended to improve participants’ intentions to consume healthier foods (34,37,40,47), dietary knowledge (37,47), and self-efficacy related to healthy food acquisition and consumption (40,47). Also, interventions positively influenced the following behaviors: fruit and vegetable purchasing (35), reducing intake of sugar-sweetened beverage (43), and reducing dietary fat intake (47).

Weight status was the only health outcome reported in the reviewed studies (n = 6, 21%) (Table 4). Each of these 6 interventions included multilevel strategies that targeted individual-level behavior change such as counseling and education, in addition to policy and environmental level change strategies that included increasing availability of healthy foods, and discouraging the consumption of sugar-sweetened beverages. Only 1 of the 6 studies reported reducing weight status of participants (36). One study reported that although children’s body mass index increased, the increase was less than in a comparison community (31). Another found that weight status increased (47), and 3 studies found that weight status did not significantly change (34,40,45).

## Discussion

We assessed the state of research on nutrition-related policy and environmental strategies for obesity prevention in rural communities. The review identified 29 studies that implemented COCOMO nutrition-related policy and environmental strategies in rural communities. Other obesity prevention reviews have typically focused on effectiveness or looked at specific populations and settings. This review included studies conducted with varied populations and settings and thus findings were too diverse to empirically assess effectiveness. Instead, our findings provide guidance on adapting and implementing policy and environmental strategies in rural communities.

In support of our a priori hypothesis, we found that many, but not all, COCOMO strategies were applied in rural settings (Table 3) and that multiple approaches were used to adapt them. The COCOMO strategies most commonly implemented in rural areas focused on increasing the availability of healthy foods and beverages and limiting the availability of unhealthy ones. Fewer studies examined approaches to limiting advertising of less healthy foods and beverages or modifying portion sizes. These findings are consistent with formative work with stakeholders in rural eastern and western North Carolina, which found that rural stakeholders rated strategies related to limiting advertising of less healthy foods and beverages as less feasible and acceptable than other COCOMO strategies (15,56). None of the studies reviewed sought to improve the geographic availability of supermarkets as recommended in strategy 3. Instead, many studies focused on improving the availability of healthier foods and beverages in small retail food outlets and increasing access to farmers markets, which may be more feasible targets for change than increasing availability of supermarkets in rural areas given the cost associated with locating supermarkets in rural areas.

### Guidance on adapting and implementing strategies in rural communities

In rural communities, policy and environmental strategies that aim to increase access to healthy foods may also promote economic development through support of farmers, retail stores, and other businesses involved in food production, distribution, and sales (57). Researchers might study strategies that locate retailer’s food outlets in closer proximity to customers, as illustrated by the use of mobile markets by Sharkey et al (58). To tailor interventions to local cultures and taste preferences, those planning rural interventions may benefit from conducting formative work to identify traditional and locally grown foods, as well as local approaches to food preparation. Formative work may also help identify local partners who may be important to promoting and implementing policy and environmental changes in rural areas.

Almost one-third of the studies (n = 10; 34%) were conducted with American Indian tribes or First Nations of Canada. Most of these studies (70%) were conducted in small retail settings (Tables 2 and 3). Research in these often under-studied, at-risk communities is critical to identifying culturally and contextually appropriate approaches to reducing nutrition-related disparities. However, tribally led nutrition-related policy and environmental strategies to prevent obesity may not be generalizable to other rural communities because of tribal governments’ authority to determine their own governance structures, pass laws, and enforce laws through police departments and tribal courts (59). More research can enhance our understanding of the role of tribal self-governance for nutrition-related policy and environmental strategies to prevent obesity (60).

Our aim was to obtain a broad picture of nutrition-related policy and environmental strategies to prevent obesity in rural communities to identify gaps and guide future research. Efforts were made to identify all relevant studies. Formative, process, and outcome evaluation studies were identified for this review, which limited our ability to compare findings across studies, as did what data were collected and reported. Many of the studies were formative. Those studies that assessed outcomes typically involved only a small number of settings and were often quasi-experimental in design. Furthermore, as with all reviews, the study was constrained by limitations in the existing literature and publication bias. Only a limited amount of research on nutrition-related policy and environmental strategies for obesity prevention in rural areas has been published in peer-reviewed journals. The authors recommend consulting websites, gray literature, and other forms of reporting for additional insight into effectiveness and implementation considerations for policy and environmental-level nutrition interventions in rural areas. Finally, we used several strategies to identify studies that were conducted in rural settings; however, studies conducted in rural areas that did not explicitly indicate that they dealt with rural settings may not have been captured in our search.

### Suggestions for future research

**Explicitly compare the effectiveness of interventions in urban and suburban settings versus rural settings.** None of the studies included in the review explicitly compared the effectiveness of policy changes in rural and urban communities. Future investigations should report observed differences in rural settings compared with other settings to inform future research aiming to reduce health disparities in rural areas. Only 14 of the 29 studies identified in this study assessed intervention outcomes at the environmental, policy, or individual level. Therefore, more work is needed to assess policy and environmental, social, psychosocial, behavioral, and biological outcomes associated with nutrition-related policy and environmental strategies.

**Experiment with a variety of intervention settings.** Among the studies reviewed, the most common settings were schools, small retail food outlets, and worksites. Additional research is needed to explore the feasibility and effectiveness of nutrition-related policy and environmental strategies in other rural settings, such as parks and recreational sites and hospitals, to identify the mix of settings that will yield the greatest population-level reach and effects.

**Explore the possibility of aligning federal food and nutrition assistance programs with efforts to increase access to local foods.** The limited research to date on COCOMO strategy 5, “improve availability of mechanisms for purchasing foods from farms,” has focused on examining the effectiveness of voucher or coupon programs through USDA. This aligns with a study conducted by the NOPREN Rural Food Access Working Group (RFAWG), examining rural stakeholders’ views about the most promising strategies for improving healthy food access in rural areas, finding that one of the highest ranked policy and research priorities included improving access to federal food and nutrition assistance programs (61).

**Report costs associated with implementing intervention strategies.** Decision-makers often need information about costs as well as effectiveness when deciding whether to invest in evidence-based nutrition-related policy and environmental strategies (62). Unfortunately, cost and cost effectiveness data are often not reported in scientific articles. In this review, 3 articles included some type of implementation cost information. Conrey et al reported the cost for implementing Women, Infants, and Children (WIC) Farmers’ Market Nutrition Program (FMNP) enhancements across New York State for one year (24); Saksvig et al mentioned that the cost of their school-based intervention was low, but did not provide specific costs (47); and Ruelle et al calculated cost distance, which is a spatial analysis technique that measures costs associated with moving across a landscape to help planners identify potential locations for farmers markets (48). When authors report cost or cost effectiveness information, decision-makers are granted important information from scientific studies that could influence their decision to adopt promising nutrition-related policy and environmental strategies.

**Explore the economic impact and the role of local champions related to increasing access to local foods.** A recent NOPREN Rural Food Access Working Group study examined rural stakeholders’ views about the most promising strategies for improving healthy food access in rural areas (61). Among the workgroup’s top recommendations was research on the economic impact that strategies have on communities as well as the implications of revenue generation and job creation on increased healthy food access and purchasing power among individuals (61). For example, policy and environmental changes that increase local market and supply chain business opportunities have potential economic benefits for agricultural communities while also increasing access to healthy foods (57). The study’s recommendations align with COCOMO strategies 5 (“improve availability of mechanisms for purchasing food from farms”) and 6 (“provide incentives for the production, distribution, and procurement of foods from local farms”). There is little available research about the effect that local champions, such as policymakers, food policy councils, and other community-driven coalitions, have on nutrition-related policy and environmental change in rural communities. A better understanding could be gained through qualitative work with community stakeholders to determine who local champions are and to identify the best ways to connect with and engage those champions.

These findings help to inform the adaption and implementation of nutrition-related policy and environmental strategies for obesity prevention in rural communities. Although our review was not able to provide policy-makers with information about the effectiveness of different policy approaches, these findings offer insights into the various options available to improve the food environment in rural communities. Moreover, decision-makers should understand the limitations of adopting strategies generated from and tested in geographically diverse settings. The findings also indicate the need for additional research. One major research gap that remains is the limited number of studies testing effectiveness of nutrition-related policy and environmental strategies in rural communities. Future work could identify strategies that have not yet been formally evaluated but that could be feasible in rural communities, such as mobile farmers markets and community garden initiatives.
